# Initial Long-Term Scenarios for COVID-19’s Impact on Aviation and Implications for Climate Policy

**DOI:** 10.1177/03611981211045067

**Published:** 2021-09-23

**Authors:** Lynnette Dray, Andreas W. Schäfer

**Affiliations:** 1 Air Transportation Systems Lab, Energy Institute, University College London, London, UK

**Keywords:** aviation, economics and forecasting, aviation forecasting, environmental impacts of aviation, climate change

## Abstract

The COVID-19 pandemic had a dramatic impact on aviation in 2020, and the industry’s future is uncertain. In this paper, we consider scenarios for recovery and ongoing demand, and discuss the implications of these scenarios for aviation emissions-related policy, including the Carbon Offsetting and Reduction Scheme for International Aviation (CORSIA) and the EU Emissions Trading Scheme (ETS). Using the Aviation Integrated Model (AIM2015), a global aviation systems model, we project how long-term demand, fleet, and emissions projections might change. Depending on recovery scenario, we project cumulative aviation fuel use to 2050 might be up to 9% below that in scenarios not including the pandemic. The majority of this difference arises from reductions in relative global income levels. Around 40% of modeled scenarios project no offset requirement in either the CORSIA pilot or first phases; however, because of its more stringent emissions baseline (based on reductions from year 2004–2006 CO_2_, rather than constant year-2019 CO_2_), the EU ETS is likely to be less affected. However, if no new policies are applied and technology developments follow historical trends, year-2050 global net aviation CO_2_ is still likely to be well above industry goals, including the goal of carbon-neutral growth from 2019, even when the demand effects of the pandemic are accounted for.

The COVID-19 pandemic has had an extreme impact on global aviation. The International Air Transport Association (IATA) estimates the global aviation industry lost over $100 billion in 2020 alone (*1*), more than three times the losses caused by the 2008 financial crisis, and that global revenue passenger-kilometers (RPK) will be lower than pre-pandemic projected levels by over 60% in 2020 and around 50% to 65% in 2021 (*2*). Impacts on aviation-dependent industries, such as tourism, are similarly large. The International Civil Aviation Organization (ICAO) estimate year-2020 global scheduled passenger numbers were reduced by 60% (*3*). Weekly European air traffic volumes were 90% below year-2019 levels in April 2020 and remain 64% below year 2019 levels in May 2021 (*4*), with Eurocontrol projections implying year-2020 traffic just below half of year-2019 values (*5*). Although 2020 RPK impacts were at a similar 60%–70% level across all world regions, 2021 IATA RPK projections anticipate more regional divergence, with faster RPK recovery in North and Latin America (40%–50% below year-2019 levels) and slower recovery in Europe and the Middle East (65%–70% below year-2019 levels) (*3*). For comparison, the 2003 SARS epidemic led to 8% yearly RPK reduction in affected regions, with 6 months of disruption, peak monthly RPK reduction of 35%, and $6 billion lost revenue (*3*).

This reduction is even more dramatic when pre-pandemic anticipated growth trends are considered. Global aviation RPK demand has grown on average over 5% per year since 1980. Industry projections anticipated 4%–5% per year increases over the next 20 years (*6*, *7*). Rapid expansion comes at the cost of increasing environmental impacts (*8*), with some studies suggesting that, because of difficulties in reducing within-sector CO_2_, aviation may become one of the highest-emitting sectors by 2050 (*9*). The challenge of reducing aviation’s environmental impact has led to policy action on national, regional, and international levels (*10*), most notably ICAO’s Carbon Offsetting and Reduction Scheme for International Aviation (CORSIA) and aviation’s inclusion in the EU Emissions Trading Scheme (ETS). In both schemes, CO_2_ emissions above a given baseline are subject to a carbon price which funds corresponding reductions in other sectors. Additionally, depending on carbon costs, airlines may decide to adopt lower-emissions technologies themselves. In response to the COVID-19 pandemic, the CORSIA baseline (initially the average of year-2019 and year-2020 eligible route emissions) has been changed to refer only to 2019 (*11*). Initial analysis has shown that this change will likely result in a zero offset requirement for the pilot phase of CORSIA and offsetting requirements to 2035 might be reduced by up to three-quarters from a no-COVID baseline, if demand recovery follows IATA projections (*12*, *13*).

Inferring ongoing impacts on aviation based on currently available pandemic recovery data and projections is not straightforward. The pandemic will affect ongoing aviation demand and emissions through multiple different channels of action, discussed in turn below. First, there are direct impacts via border closures and movement restrictions; second, indirect effects via airline bankruptcies and aircraft retirements; third, impacts of changes in fuel and/or carbon prices; fourth, changes in technology investment; fifth, changes in attitudes to flying; and sixth, the ongoing passenger and freight demand impact of reductions in economic growth. Each of these channels may affect ongoing emissions-reduction efforts and cumulative sectoral emissions. The purpose of this paper is to provide initial scenario estimates for what the long-term impacts on emissions policy and aviation emissions outcomes might be.

Airline bankruptcies and retirements may continue to affect the sector even during recovery, because the mix of aircraft and operators has changed. The initial crisis period was associated both with airline bankruptcies (e.g., Avianca, Flybe, LATAM) and scrappage and/or storage of older and larger aircraft (*14*). Historically, routes vacated by collapsed airlines were typically taken up by other airlines (*15*), suggesting networks and competition may return to pre-pandemic conditions over the longer term provided profits can still be made. Other airlines have received government bailouts to continue operating, with over $100 billion state aid globally for aviation industry-related entities (*16*). Aircraft storage is a common response to demand downturns. Although some aircraft are retired from storage, others resume service once demand increases again (*17*). For very large and/or quad-engine aircraft such as the Boeing 747, Airbus A380, and Airbus A340, increased storage may combine with the end of production runs to accelerate existing system trends away from their use. Increased financial constraints on airlines may also incentivize abandonment of sustainability measures (*18*).

Oil prices in 2020 stabilized at around $40/bbl, well below pre-pandemic projections of around $60/bbl, but have recovered in 2021 (*19*). Reductions in oil price are typically passed on to passengers via lower ticket prices, stimulating demand; however, because many airlines hedge fuel costs, this effect may have a lag of 1–2 years (*20*). The short duration of the oil price fall means that any ongoing impacts are likely to be small. Low oil prices combined with capital constraints may also reduce airline incentives to invest in fuel-saving technologies. Historically, low oil price has been associated with lower ambition in fuel-saving technology development, although conditional bail-out programs for affected industries (e.g., the French government’s Airbus aid package [*21*]) may mitigate this. The impact of COVID-19 on carbon prices has been smaller. Initially, the EU ETS European Union Allowance (EUA) price fell by around 30% (*22*), but has since recovered to above pre-pandemic levels, driven in part by the EU ETS Market Stability Reserve (*23*). However, longer-term effects on EUA prices are possible if lower projected gross domestic product (GDP) reduces energy demand. Similarly, international carbon credit (CER) prices remain around recent levels under $1/tCO_2_ (*23*).

Longer-term changes in attitudes to aviation arising from the pandemic could occur because flying is perceived as less safe, or from increased use of videoconferencing to substitute business travel. Although demand downturns caused by previous disruptive events associated with ongoing safety concerns (e.g., the terrorist attacks of September 11, 2001 and the 2003 SARS epidemic) were relatively short-lived, there is evidence of longer-term impacts on post-9/11 US domestic demand (*24*). This is an area of considerable uncertainty and potentially high impact. As yet it is impossible to quantify long-term impacts on business travel, though there are early indications that reductions are likely (*25*). Business passengers were around 30% of year-2007 UK totals, and this proportion has been declining over time (*26*). Separately, it has been suggested that aviation demand growth may decouple from GDP growth as aviation systems mature and/or because of environmental campaigns such as the flight-shaming movement, which encourage individuals to fly less (*27*). However, a comparison of income elasticities of aviation demand from different world regions suggests systematic differences between more and less mature aviation systems are not straightforwardly identifiable with current estimates (*28*).

Historically, aviation demand has been sensitive to developments in global incomes (*28*). Although demand impacts caused by border closures, movement restrictions, and fear of contagion may be relatively short-lived, the income impacts of the pandemic are likely to be longer term. The April 2021 update to the International Monetary Fund (IMF)’s World Economic Outlook (*29*) estimates that global GDP fell 3.3% in 2020 and will recover by 6% in 2021. Similarly, the World Bank (*30*) estimates a 4.3% fall in year-2020 global GDP and recovery of 4% in 2021. Longer-term projections are necessarily based on scenarios for how the pandemic progresses; however, ongoing offsets in GDP per capita from pre-pandemic projections are typical. This is similar to the impact of the 2008 financial crisis; IMF analysis (*31*) suggests that, 10 years later, 85% of economies that experienced a banking crisis in 2008 were still operating at output levels below pre-crisis trends. IMF estimates (*29*) suggest that global GDP will remain 2%–4.5% lower than pre-pandemic projections in 2025. Aviation passenger and freight demand can be strongly affected by changes in income (*28*), so it is likely that ongoing offsets in GDP projections will also affect long-term aviation projections; for example, the 2008 financial crisis was associated with a 2-year demand offset from pre-crisis trends (*12*). Because ongoing GDP impacts are likely to differ by country and will be affected by factors such as vaccine availability and the extent and type of outbreak control measures, ongoing demand impacts will likely differ by country as well.

Each area of impact (movement restrictions; bankruptcies and retirements; changes in fuel prices and technology investment; changes in attitudes to flying; and offsets in GDP/capita), may affect aviation demand, fleet, and emissions over long timescales, affecting the likelihood of reaching emissions targets and the functioning of emissions-reduction policy. In this paper, we use a global aviation systems model to investigate scenarios for how the post-COVID-19 aviation sector might develop to 2050, and what implications these scenarios have for aviation emissions policies, both in specific cases (e.g., the EU ETS and CORSIA) and more generally in relation to cumulative CO_2_ budgets.

## Methods

### Modeling

**Figure 1.**
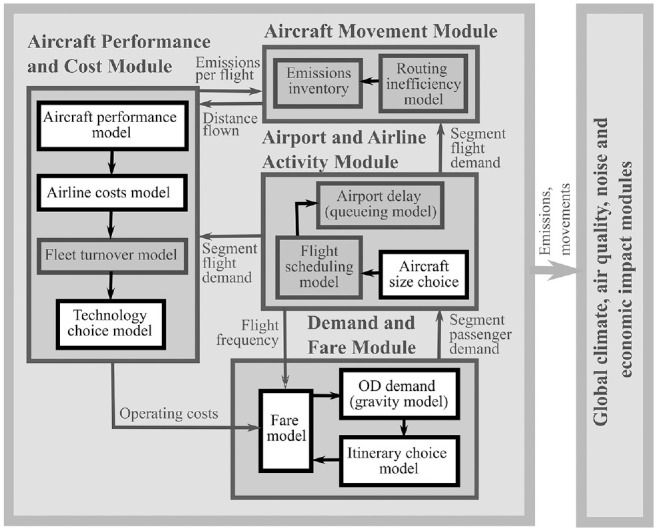
Aviation integrated model (AIM2015) structure. *Note*: OD = origin–destination.

To project aviation demand and emissions with and without the COVID-19 pandemic, we use an updated version of the open-source global aviation systems model AIM2015 (*28*). AIM2015 models current and future interactions between passengers, airlines, airports, and other stakeholders. The basic model structure is shown in Figure 1. Passenger demand between cities is projected using a gravity model based on population, income, and fare, and distributed between airports and routes using an itinerary choice model. An aircraft size choice model projects which aircraft are used on each route; estimated load factors are then used to project flight schedules, demand for aircraft, and delays. Based on costs, airlines choose technologies and fuel-saving measures to adopt. The segment costs to airlines of flying this schedule with the given fleet at a given fuel and carbon price are estimated and input to fare modeling, which feeds back into demand and itinerary choice calculations. When equilibrium is reached between supply and demand, output metrics are calculated. The structure and validation of these sub-models and of the overall model is discussed extensively elsewhere (*28*).

Several additions have been applied to more accurately model the impact of COVID-19 on demand, fleets, and emissions, and interactions with aviation policy. First, because CORSIA and the EU ETS apply to emissions totals above an absolute baseline, a full assessment requires modeling all sources of eligible aviation CO_2_. Therefore the model has been adapted to include simple models for unscheduled passenger flights and air freight. Second, fleet modeling has been adapted to account for the phasing out of very large and/or quad-engine aircraft, which has been accelerated by the COVID-19 pandemic. Third, a demand damping variable to cover the impact of movement restrictions has been introduced. These areas are discussed individually, below.

#### Unscheduled Passenger Flights

Unscheduled flights consist mainly of charter flights and on-demand air services (e.g., commercial business flights). In 2015, they accounted for 217 billion RPK, or 5% of global international RPK, although this proportion is declining (*32*). Historically, Europe accounted for over 90% of global non-scheduled traffic; a “typical” charter flight travels between a European regional airport and a resort (*33*). Several papers and reports provide information about size distributions and typical proportion of unscheduled flights by region and type of origin and destination airport (*32*–*38*). These are used to generate scaling factors per route type (e.g., international routes from regional UK airports) to account for additional unscheduled flights.

#### Air Freight

Air freight accounted for around 24% of global RTK in 2018 (*32*). This includes freight carried in freighter aircraft and in the holds of passenger aircraft (roughly 50% of total freight RTK [*39*]). Less information is available about air freight than is available for passengers. For this paper, we follow the same simple approach as in ICF Consulting et al. (*40*) to provide an estimate of air freight-related emissions. 2015 base year country-pair air freight flows are derived from national and international databases where available (*41*) and otherwise estimated from country-level totals combined with typical hold freight flows (*32*, *42*). For hold freight, the constraint on available capacity is often volume rather than weight. As such, we assume that typical passenger-to-freight ratios in passenger aircraft per route group (*42*) represent what is practically achievable, and that additional freight per country-pair flow beyond what can be carried in passenger aircraft at these ratios is carried in freighter aircraft. Freighter fleets and utilization are derived from fleet databases (*43*) and freight-specific operating costs are taken from literature estimates (*44*). The technology composition of the freighter fleet is assumed similar to that for passenger aircraft of the same size and manufacture year. Freighter conversion from passenger aircraft is accounted for by conversion curves (*45*). Changes in air freight demand resulting from changes in country-level GDP and country-pair level operating costs are accounted for via literature elasticity estimates (*46*). Freighter flights are not assigned to individual airports but aggregated to country-pair level, with flight distance assumed typical of the average flight between each country-pair. For the pandemic period, where extra freighter flights are required as a result of the reduction in passenger aircraft hold freight capacity, it is assumed they will be provided by stored freighter aircraft (if available) or otherwise by stored passenger aircraft with technology characteristics typical of the passenger fleet, approximating the case where passenger aircraft have been used to provide all-freight operations (*47*). This approach is calibrated by a similar backcasting exercise to that carried out in Dray et al. (*28*), and, although approximate, produces similar freight RTK, demand growth rates, and cost/RTK estimates to industry projections (*39*). We neglect transhipment-related and airport-specific effects and interactions with other transport modes.

#### Aircraft Retirements and Very Large Aircraft End of Production

Storage of aircraft during demand downturns (and either retirement from storage if demand remains low, or stored aircraft returning to operations in the case of demand recovery) is already modeled in AIM. However, as discussed above, the COVID-19 pandemic has likely accelerated existing trends away from Very Large Aircraft (VLA). The Airbus A380 and Boeing 747 are due to end production before 2023, with no scheduled replacement. To simulate this, we restrict airline purchases and modeled technology development in the VLA size class from 2021 onward; as the fleet declines through retirements, demand that would have been served by VLAs is typically served by twin-aisle aircraft at higher frequency but lower per-flight CO_2_. However, the overall impact of this change over a case where VLA sales and development continue is small—around a 0.3% decrease in global direct aviation CO_2_ by 2035.

#### Border Closures

The largest short-term impact of the pandemic on aviation arises from travel restrictions. International RPK was 98% below year-2019 levels in May 2020 (*1*), driven by border closures, quarantine regulations, and distancing requirements. It is likely some movement restrictions will extend into late 2021 and potentially beyond, depending how the pandemic progresses. The extent and duration of these effects is highly uncertain. To generate scenarios for these impacts, we introduce demand damping factors for the immediate recovery period, applied separately for domestic and international passenger and freight demand. For 2020, these factors are estimated directly from available data on yearly average demand reductions (*1*, *3*, *5*). After 2020, we follow a scenario-based approach where the assumed extent of movement restrictions is consistent with the recovery scenario, as discussed below.

Passenger damping factors apply to numbers of true origin–ultimate destination passengers per city-pair. In practice, flights are reduced by a smaller amount, with many operating at low load factors (*1*). To account for this, passenger load factors are also adjusted when the damping factor is applied, with the amount of adjustment taken from IATA data on typical load factors for routes with strongly reduced demand (*1*). Because airlines lose money operating at low load factors, this is not sustainable over the long term and we assume that, when the damping factor is removed, load factors will return to pre-pandemic trends.

### Future Scenarios

As discussed above, the main long-term impacts of COVID-19 on aviation are likely to come from changes in income and energy prices; from fleet storage and retirements; from changes in technology investment; and, potentially, from changes in attitudes to aviation. Over a shorter time period, physical restrictions on travel will also affect demand. These developments remain highly uncertain. Separately, ongoing trends in population, income, and energy prices remain uncertain even in scenarios where the effect of the pandemic is not modeled.

**Figure 2.**
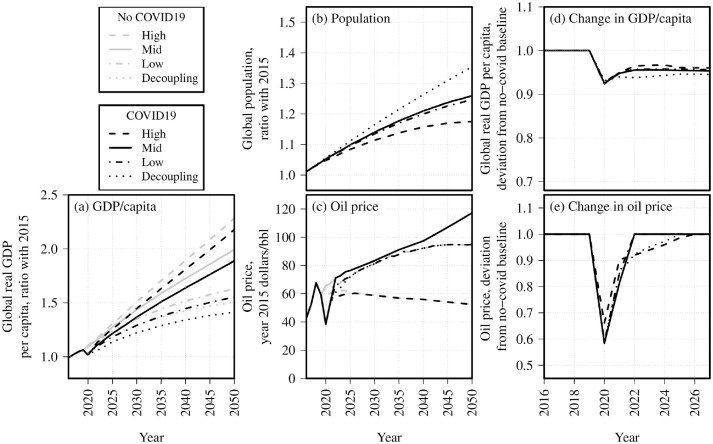
Scenarios for development of (*a*) global gross domestic product (GDP)/capita, (*b*) population, and (*c*) oil price to 2050, with and without COVID-19, and variation in GDP/capita and oil price from the corresponding no-COVID-19 scenario, 2018 to 2025.

To address these uncertainties, we use two approaches. For the main set of model runs, we use a scenario-based approach to define internally consistent sets of model inputs. Three scenario components are required. First, long-term population and income growth rates are derived from the Intergovernmental Panel on Climate Change (IPCC) Shared Socioeconomic Pathway (SSP) scenario set (*48*). As in Dray et al. (*28*), we consider SSP1, 2, and 4 as High-growth, Mid-growth, and Low-growth scenarios. These scenarios are paired with long-term energy price scenarios consistent with their broad storylines: the International Energy Agency (IEA) Sustainable Development Scenario (SDS) and Stated Policies Scenario (STEPS) for High (SSP1) and Mid (SSP2), and Energy Information Administration (EIA) “low economic growth” projections for Low (SSP4 [*49*, *50*]). However, to explore an illustrative case where changes in attitudes to aviation might cause significantly less demand growth than projected, we also run IPCC’s very low economic growth case SSP3 with an additional demand decoupling component (“Decoupling” scenario), using decreases in income elasticities over time similar to Department for Transport (*51*). Second, modeling the impact of COVID-19 on aviation requires projections for how the pandemic will affect GDP and energy prices. IMF provides country-level projections for 2020 and 2021, and global scenarios for how developments to 2025 may deviate from a no-pandemic baseline (*29*). We run the four scenarios with and without COVID-19 impacts. For the scenario runs which include COVID-19, we adjust GDP and oil prices between 2020 and 2025 in line with the projections of IMF (*29*), using actual values for 2020 and differences from a no-pandemic baseline for later years, and running the “High” long-term growth scenario with the IMF’s “Upside” scenario and the “Decoupling” scenario with the IMF’s “Downside” scenario (*29*). These combinations are not intended to imply that individual recovery scenarios are likely to lead to specific ongoing GDP scenarios, but rather an attempt to cover the range of potential futures (from “rapid recovery followed by high growth” to “prolonged recovery followed by weak growth”) which may be relevant for aviation policy outcomes. Model inputs per scenario with and without COVID-19 are shown in Figure 2 and are summarized in Table 1.

**Table 1.** Summary of Input Assumptions Across the Different Scenarios

Variable	Scenario					Sources
	High	Mid	Low	Decoupling	MC	
Population scenario	SSP1	SSP2	SSP4	SSP3	UN WPP	UN (*54*) (historical values); UN, O’Neill et al. (*48*, *53*) (projections)
Year 2050 global population, ratio with 2015	1.18	1.26	1.25	1.36	1.30 (1.24–1.37)^a^	
Baseline GDP/capita scenario	SSP1	SSP2	SSP4	SSP3	Christensen et al (*53*).	UN (*54*) (historical values); IMF (*29)* (pandemic) O’Neill et al., Christensen et al. (*48*, *53*) (projections)
COVID-19 economic impact scenario	Upside	Baseline	Baseline	Downside	Upside–Downside	
Year 2050 global GDP/capita, ratio with 2015	2.18 (2.28)^b^	1.89 (1.99)	1.55 (1.63)	1.41 (1.50)	1.87 (1.18–3.03)^a^	
Oil price scenario	IEA SDS	IEA STEPS	EIA Low Growth	EIA Low Growth	IEA SDS–IEA STEPS	IEA, EIA (*49*, *50*)
Year 2050 oil price, Year 2015 dollars/bbl	52	117	95	95	52–117	
Movement restriction scenario based on:	IATA upper	IATA baseline	IATA baseline	IATA lower	IATA upper–lower	IATA (*2*) (pre-2025 only)
Demand decoupling from GDP/capita growth	No	No	No	Yes	No	(*51*) (where used)
EU ETS carbon price, 2050, year 2015 dollars/tCO_2_	146	146	146	146	146–430	(*58*)
CORSIA carbon price, 2050, year 2015 dollars/tCO_2_	1.2	1.2	1.2	1.2	1.2–12.0	(*60*)

a The median value across all runs is shown with interdecile range given in brackets.

b The value for the no-COVID-19 scenario is shown in brackets. Note that differences between the COVID19 and no-COVID-19 scenarios are not identical to those examined in IMF (*29*) because the different baseline scenarios have different assumptions about growth over the 2020 to 2021 period.

Passenger damping factors to account for closed connections for 2020 and 2021 are derived from IATA data and projections (*2*); for SSP3, we additionally extend applied damping factors to 2025 to simulate a prolonged movement restriction scenario based on the low end of IATA short-term impact projections (*2*). Air freight demand has reduced by much less than passenger demand. For 2020 a drop in global trade volumes of 13% to 32% has been projected (*3*). Because hold freight capacity in passenger aircraft has dropped by a larger factor than this, COVID-19 has driven an effective short-term shift toward freighter aircraft, with some passenger aircraft temporarily repurposed for freight-only flights (*52*), and we apply damping factors to freight demand for 2020 only.

Scenario modeling allows the development of self-consistent storylines about how the future may develop. However, it may miss future developments which are within the uncertainty range in developments in different parameters, but do not form part of the specific storylines investigated. In general, GDP/capita is the scenario variable which has the largest impact on aviation demand. Recent research has suggested the SSP scenarios may understate the full range of uncertainty in future GDP developments (*53*). To assess how uncertainties in GDP, population, and energy prices may affect outcomes, we also run a set of 500 model runs with global GDP per capita growth rates drawn from distributions estimated by Department for Transport (*51*), including a range of COVID-19 GDP adjustments between the “Upside” and “Downside” scenarios from IMF (*29*). We also include population growth rate distributions from United Nations (*54*), although these have relatively little effect on outcomes because of the smaller uncertainty range in population growth rates and lower model sensitivity (for example, running the SSP2 GDP/capita growth scenario with SSP1 or SSP3 population growth rates changes year-2050 RPK by only ±3%).

**Figure 3.**
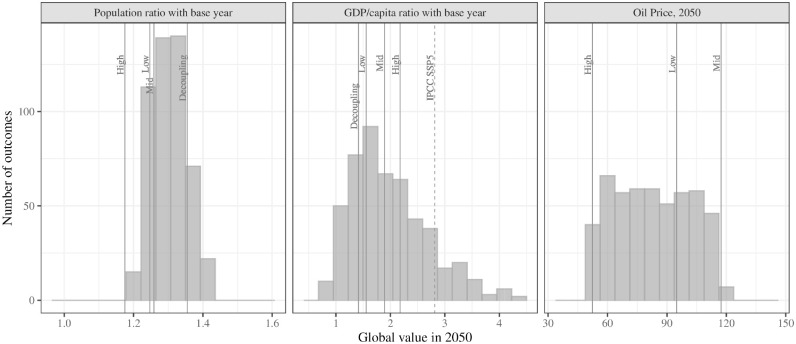
Distribution of year-2050 outcomes for model runs with probabilistic inputs, in comparison to scenario-based values. Note that scenario names shown refer to aviation growth rates rather than trends in individual variables.

For these projections, oil prices and pandemic period damping factors are drawn from a uniform distribution across the full range of those modeled in the scenario-based projections. Year-2050 distributions of GDP/capita, population, and oil price for these model runs are shown in Figure 3, compared with scenario values. Because uncertainty in GDP/capita growth rates rather than absolute values is modeled, GDP/capita distributions are not symmetric, with a long tail of high-GDP outcomes as noted in Christensen et al. (*53*). For reference, the IPCC very high (SSP5) growth scenario is also shown for GDP/capita.

#### Modeling CORSIA and the EU ETS

The EU ETS currently applies to intra-European Economic Area (EEA) flights. An aviation emissions baseline is set corresponding to 95% of average year 2004–2006 aviation CO_2_ (after adjustments for change of scope, around 38.7 Mt in 2019; [*55*]). EU Aviation sector Allowances (EUAAs) are allocated to cover emissions below this baseline, 85% of which are freely allocated via benchmarking or the new entrants’ reserve, and 15% of which are auctioned. Emissions above the baseline must be accounted for via the purchase of allowances (EUAs) from other sectors. The aviation cap will decrease by a Linear Reduction Factor (LRF) of 2.2% per year from 2021 to 2030, in line with other ETS sectors (*56*), though the LRF may be increased as part of the “Fit for 55” package (*57*). In 2019, around 48% of EU ETS-eligible direct aviation CO_2_ was above the baseline. This means that a 50% reduction in aviation demand at constant aircraft utilization by type and load factor would likely take emissions below the baseline. However, both aircraft utilization by type and load factors have changed substantially. The future scope of the scheme, as well as the continuation of the LRF past 2030 and free allowance allocation, are under regular review and may be subject to change (*40*). For this analysis, we assume that the EU ETS continues to apply to intra-EEA flights (with the addition of a link to the Swiss ETS; note that the intra-EEA scope means that the UK is assumed to leave the scheme and the UK ETS which will replace it is not modeled here as its details are still under development) and that the same LRF and auctioning percentage continue to apply through to 2050.

For CORSIA, global international aviation between participating countries was initially intended to be offset above an average year 2019–2020 direct aviation CO_2_ baseline. This has recently been adjusted to a year-2019 baseline only (*11*). As noted by several authors (*12*, *13*), a 2019 baseline would imply no offsets under CORSIA until demand recovers, potentially leading to zero offset requirements in the pilot and/or first phases of the scheme. Initially, all airlines on eligible routes pay carbon costs under CORSIA corresponding to the amount by which total scheme emissions are above the baseline. From 2030 on, the scheme shifts to a combination of global and individual airline responsibility for growth in emissions. This shift effectively lowers the baseline slightly, as under individual responsibility airlines that can reduce their own emissions below the baseline have zero offset requirements rather than lowering the offset requirement for others. We assume participation throughout all phases of the scheme is limited to those countries that have indicated to ICAO that they will participate, that is, excluding India, China, Brazil, Russia, and Vietnam, and that the scheme is continued to 2050.

Different carbon prices apply to both schemes. Because the EU ETS is a carbon trading scheme, EU ETS carbon prices are a function of the cost of emissions reductions achievable across participating sectors and geographical regions. They are therefore sensitive to assumptions about the future level of ambition of the scheme and technical implementation details such as the Market Stability Reserve. As a baseline, we use carbon price projections from European Commission (*58*) (reaching around $150/tCO_2_ by 2050); following Department of Energy & Climate Change (*59*), when a range of carbon prices is used, we consider values beween the baseline and a higher trend reaching the upper-end values modeled in European Commission (*58*) in 2050 (around $430/tCO_2_).

CORSIA Eligible Unit (CEU) prices are likely to reflect the cost of international carbon credits (CERs). Fearnehough et al. project that the cost of CER-type credits is likely to remain under €1/tCO_2_ ($1.2/tCO_2_) for the pilot and first phases of CORSIA, with the potential to rise to €4–10/tCO_2_ ($5–12/tCO_2_) if additional constraints on vintage or vulnerability are applied (*60*). We use this lower price trend as a baseline and the upper price trend as an upper limit where a range of values is modeled. However, because the CORSIA carbon price applies only to emissions above the baseline, the effective carbon price that airlines will pay is much lower than this, and the exact value chosen for CORSIA carbon price has little impact on outcomes.

#### Technology Characteristics

The technological makeup of the global aircraft fleet in 2050 is uncertain. Because the typical lifetime of an aircraft to scrappage is 30 years, in the absence of a change in retirement behavior many aircraft delivered in 2020 will still be operating in 2050 (*45*). The next generation of aircraft designs is expected in the 2030–2035 time period (*61*) and these designs will likely dominate the year-2050 fleet. Although substantial research is ongoing into alternatives to conventional aircraft, including electric and hydrogen-fueled concepts, technology and infrastructure constraints likely mean that the full global potential of these designs is realizable only after 2050 without additional policy support; where literature estimates project significant aviation decarbonization before this date, this is typically assumed to arise via low-carbon drop-in fuels (either biofuels or power-to-liquids) or via offsets in other sectors (*9*, *62*). For this paper, we assume “business-as-usual”-type technology developments, that is, a kerosene-fueled next generation of aircraft, using technology and cost characteristics from ATA & Ellondee (*61*). For the main scenario runs, central values of these characteristics are used; where distributions of input values are assumed, we use triangular distributions bounded by the “optimistic” and “pessimistic” scenarios provided. Following Climate Change Committee (*9*), we assume up to 10% aviation biofuel use, with cost characteristics given by the cost curve model used in Dray et al. (*63*). The impact on outcomes of deviating from these (relatively conservative) technology assumptions are discussed below.

## Results and Discussion

### Scenario-Based Modeling

**Figure 4.**
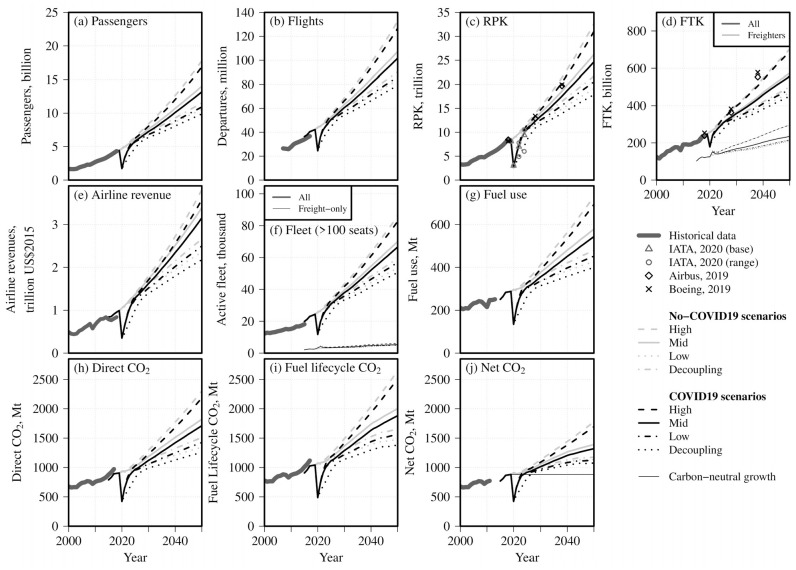
Key system output metrics to 2050 from scenario modeling, COVID-19-adjusted (*black lines*) and non-COVID-19-adjusted (*gray lines*) model runs. Panels: (a) passengers; (b) flights; (c) revenue passenger-km (RPK; (d) freight tonne-km (FTK); (e) airline revenue; (f) airline fleets; (g) fuel use; (h) direct CO_2_ emissions; (i) fuel lifecycle CO_2_ emissions; (j) net CO_2_ adjusted for allowances and offsets.

A summary of key global output metrics to 2050 with and without COVID-19 adjustment is shown in Figure 4. Historical data from ICAO, Flightglobal, and IEA (*32*, *43, 64*; note that historical fuel and CO_2_ totals shown include military flights), pre-COVID-19 industry projections (*6*, *7*) and alternative post-COVID-19 projections (*2*) are also shown. Black lines indicate COVID-19-adjusted scenarios; gray lines the corresponding scenarios without adjustment. For the final panel, (*j*), the level of net CO_2_ emissions consistent with carbon-neutral growth from 2019 (across both domestic and international flights) is also shown. Because we assume a range of recovery scenarios, one impact of COVID-19 is an increase in the uncertainty range of demand and emissions outcomes. This in turn increases the challenges associated with planning for system recovery and future growth. However, all combinations of scenarios have year-2050 demand and emissions below those in the corresponding no-COVID-19 case, reflecting assumed offsets in economic growth. For the three main scenarios, which do not consider demand decoupling from GDP growth, RPK demand growth in the 2030–2050 period returns to values not far below recent growth trends (2.5%–3.8%/year). For the Decoupling scenario, which includes slow recovery, slow growth, and modest demand decoupling from economic growth, RPK grows in this period at 2.2%/year, a rate at which improvements in conventional technology could feasibly be fast enough for global CO_2_ emissions growth to be close to zero (*63*). Underlying growth rates and pandemic impacts vary by country and region. For example, scenario projections anticipate average year 2019–2050 RPK growth of 3.4%–5.1% in China (3.5%–5.2% without the impact of COVID-19), 2.0%–3.5% in the United States (2.1%–3.6% without the impact of COVID-19), and 2.5%–4.6% in Brazil (2.8%–4.8% without the impact of COVID-19).

Over the short term, the largest impacts on aviation demand and emissions arise from border closures and other movement restrictions; these affect international passenger totals and RPK most strongly. Flights and fuel use are less affected owing to reductions in passenger load factor flown and owing to freighter-related components. Similarly, decreases in passenger load factor temporarily increase CO_2_ per RPK flown; although this is also affected by changes in fleet composition and route type, the overall short-term impact on CO_2_/RPK that we project is an increase (by around 6%–19% under the assumptions used here, although this is highly uncertain). Freight tonne-km (FTK) decreases by a relatively small amount, but the proportion of freight flown in freighter aircraft increases significantly over the 2020–2021 period before returning to more typical historical values.

Of the different mechanisms discussed above by which the pandemic may affect long-term aviation demand and emissions, by far the largest direct impact comes from assumed offsets in economic growth leading to lower demand. By 2050, this channel of impact is responsible for nearly all differences between the COVID-19 and corresponding non-COVID-19 cases. In contrast, fuel burn impacts related to aircraft retirements during the pandemic are typically small and/or short-lived; over the longer term, there are small differences in fleet fuel efficiency compared with the corresponding non-COVID-19 cases as a result of a combination of lower demand growth leading to slower fleet turnover, reduced pandemic period demand for new aircraft displacing some new aircraft purchases to later dates, and small changes in aircraft size choice. These factors lead in combination to 0.5%–0.8% lower year-2050 fleet fuel use per revenue tonne-km (RTK) in the COVID-19 scenarios. By 2050, global projected direct aviation CO_2_ in the COVID-19-adjusted scenarios is 1260–2180 Mt, which is around 5%–7% below the corresponding non-COVID-19 cases.

### Implications for Policy

**Figure 5.**
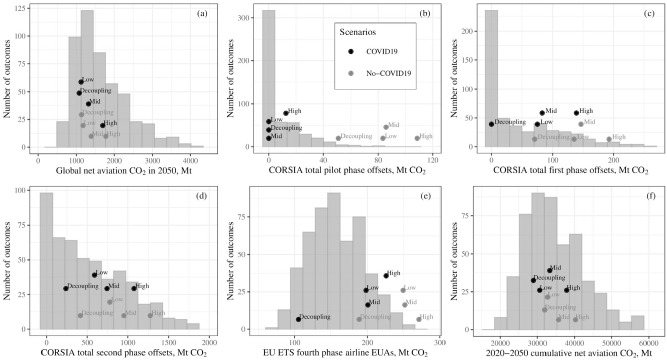
Aviation policy-related metrics. (*a*) global year-2050 net aviation CO_2_: (*b*)–(*d*) CORSIA total offsets by phase, (*e*) EU ETS fourth phase airline EUA purchases from other sectors, and (*f*) cumulative net 2020 to 2050 aviation CO_2_, for scenario model runs (points) and using probabilistic inputs (bars).

Figure 4 shows scenario-based net aviation CO_2_ including the effect of offsets and allowances purchased from other sectors (panel [*j*]), under the assumption that CORSIA offsets equate to real, additional, and permanent reductions in CO_2_. This represents a lower limit in net climate impact as, historically, CERs have been of only limited additionality (*65*)—however, CORSIA-eligible credits have additional requirements designed to improve this situation. Figure 5 shows a range of policy-relevant metrics for both scenario-based model runs and runs with probabilistic inputs. Output metrics for both sets of runs are summarized in Table 2. This includes global net aviation CO_2_ in 2050 under the “business-as-usual” policy and technology assumptions used here (i.e., CORSIA continues at current scope after 2035; the EU ETS aviation cap continues to reduce after 2030 at constant LRF; and that there is not widespread use of radically different aircraft technologies before 2050; panel [*a*]).

**Table 2.** Summary of Outputs Across the Different Scenarios

Variable	Scenario				
	High	Mid	Low	Decoupling	MC
Year 2050 global aviation tonne-km, trillion	3.79 (3.97)^a^	3.02 (3.19)	2.54 (2.67)	2.23 (2.37)	3.10 (1.77–5.62)^b^
Year 2050 global direct aviation CO_2_, Mt	2180 (2300)	1710 (1820)	1420 (1510)	1260 (1340)	1780 (1010–3320)
Year 2050 global net aviation CO_2_, Mt^c^	1700 (1780)	1320 (1390)	1120 (1180)	1080 (1130)	1460 (934–2570)
Pilot phase CORSIA offsets, MtCO_2_	13 (109)	0 (86)	0 (83)	0 (51)	0 (0–28)
First phase CORSIA offsets, MtCO_2_	139 (193)	83 (146)	75 (135)	0 (71)	13 (0–138)
Second phase CORSIA offsets, MtCO_2_	1080 (1270)	743 (951)	778 (589)	237 (415)	426 (10–1190)
EU ETS fourth phase EUAs purchased by airlines from other sectors, MtCO_2_	226 (271)	200 (251)	198 (249)	105 (189)	155 (110–207)
2020–2030 cumulative net aviation CO_2_, Mt	9,830 (11,000)	9,380 (10,600)	9,200 (10,300)	8,320 (10,200)	9,010 (7,920–10,100)
2020–2050 cumulative net aviation CO_2_, Mt	37,900 (40,200)	33,200 (35,700)	30,700 (32,700)	29,000 (32,000)	33,300 (25,600–46,000)

*Note:* CORSIA= Carbon Offsetting and Reduction Scheme for International Aviation; EU ETS= European Union Emissions Trading Scheme; EUA= European Emissions Allowance.

a Scenarios including COVID-19 shown, scenarios excluding COVID-19 given in brackets.

b The median value across all runs is shown with interdecile range given in brackets. All MC runs include the impact of COVID-19.

c Net aviation CO_2_ subtracts CORSIA offsets, EU ETS allowances purchased from other sectors and reductions in CO_2_ related to the fuel lifecycle component of aviation alternative fuel use.

Similarly to ICCT (*42*), we project year-2019 global direct aviation CO_2_ of just over 900 Mt. Long-term net global aviation CO_2_ remains above this value in 91% of model runs, even once the impact of CORSIA and the EU ETS is considered. This reflects several factors. First, CORSIA excludes domestic aviation, so rises in domestic aviation CO_2_ (excluding EU ETS participants) are not subject to offsets. Second, CORSIA covers international routes between participating countries, so non-participation of individual states excludes all flights to and from those states. Although the EU ETS includes domestic flights and has a more stringent target, it covers only around 7% of global aviation CO_2_ at current scope. Third, under most combinations of input parameters long-term demand continues to grow. For the Decoupling scenario, year-2050 net aviation CO_2_ is only around 20% above year-2019 values, and at the low end of the model runs using probabilistic inputs, net aviation CO_2_ remains around or slightly below year-2019 values. None of the combinations of parameters investigated results in IATA’s target of year-2050 aviation CO_2_ emissions being half of year-2005 values (i.e., around 325 MtCO_2_) being met, with the lowest projected year-2050 net aviation CO_2_ being around 600 Mt. Meeting this goal would require significant changes in technology and/or aviation fuels, or significantly more stringent offset-related policy (for example, a much lower CORSIA baseline, higher CORSIA participation, and action on domestic flights). As noted by Department for Transport (*51*), there is also a “long tail” of high emissions outcomes where long-term economic growth rates after the recovery period are on the upper end of those projected. As discussed above, the main impact of the COVID-19 pandemic on meeting year-2050 emissions targets is likely to be the extent to which the pandemic offsets or reduces global demand trends. In the case that economic recovery is rapid and attitudes to aviation do not change (e.g., in the High-growth scenario), the pandemic has limited impact on year-2050 emissions.

In panels (*b*)–(*d*) of Figure 5, we estimate offset totals under CORSIA for the three phases of the scheme: pilot (2021–2023), first (2024–2026), and second (2027–2035). For comparison, Healy projects (pre-COVID-19) totals across the three phases, assuming relatively rapid demand growth and that China participates throughout, of 1637–2732 Mt (*66*). Because of different demand growth and participation assumptions, our estimated totals are typically lower than this, though we see similar offset totals (up to around 2380 Mt) in the long upper tail of probabilistic model runs. Offset totals are highly sensitive to demand growth and, to a lesser extent, technology characteristics. Under three of four COVID-19 scenarios investigated, and for 58% of the COVID-19 model runs with probabilistic inputs, we find zero or close to zero offsetting requirement during the pilot phase. For the Decoupling scenario, and for 37% of probabilistic COVID-19 scenarios, we find zero offsetting requirement in the CORSIA first phase as well, that is, CORSIA scope emissions remain below year-2019 values in these scenarios until at least 2026. For CORSIA’s second phase, all investigated scenarios have at least some offsetting requirement; this is driven by the switch toward individual rather than global-level offsetting requirements. However, 23% of probabilistic model runs have total second-phase offsetting requirements below 100 Mt—well below pre-COVID-19, high-participation, high-growth projections. Under these circumstances, CORSIA would have little impact on global aviation emissions, with slowing of net aviation CO_2_ growth driven largely by demand growth slowing relative to the rate of technological improvements.

We project that European aviation CO_2_ will likely be below the EU ETS aviation cap in 2020. In the case of a prolonged second wave scenario, emissions may also be close to the cap in 2021–2022, although this is less likely as it requires prolonged intra-European flight restrictions. Over the longer term, we project EU ETS aviation CO_2_ returning to above-cap values and remaining there. This reflects both the relative stringency of the EU ETS aviation cap compared with the CORSIA baseline, and planned yearly cap reductions. Year 2019–2030 intra-EEA RPK grows by an average of 1.4%–4.5%/year across the different scenarios (1.7%–4.8%/year for scenarios without COVID-19) but, owing primarily to the EU ETS, net intra-EEA aviation CO_2_ decreases on average by 1.7%–2.5%/year over this time period (1.6%–2.4%/year for scenarios without COVID-19). Panel (*e*) of Figure 5 shows total aviation allowance purchases from other sectors (i.e., EUAs) during the scheme’s fourth phase (2021–2030). Although including the impact of COVID-19 reduces the requirement for allowances from other sectors over this time period (by around 17%–44% depending on recovery scenario), demand for allowances is much less uncertain than CORSIA offset demand. In 2030, in scenarios with low demand growth and/or extended recovery, the number of EU ETS allowances purchased by aviation from other sectors exceeds the number of global CORSIA offsets despite the latter scheme’s much larger scope.

Finally, panel (*f*) of Figure 5 shows absolute 2020–2050 global cumulative net aviation emissions. Over the long term, the climate impact of aviation is a function of cumulative emissions rather than target year values (*67*). Using this metric, we project reductions in cumulative net aviation CO_2_ by around 6%–9% from the no-COVID-19 case for the scenario model runs, depending primarily on the speed and extent of long-term economic recovery. Although none of the scenarios modeled here are consistent with net zero in the aviation sector (which would require much more significant policy and/or technological change), these reductions may still make emissions reductions that depend on finite resources (for example, limited biomass supplies) easier to achieve.

## Conclusions

In this paper, we used systems modeling to generate initial scenarios for the long-term impacts the COVID-19 pandemic may have on aviation, in particular on emissions mitigation policy. Although outcomes are highly uncertain, both scenario-based modeling and projections using probabilistic inputs suggest some long-term impacts are likely. We find that long-term impacts from pandemic-induced fleet changes are likely small, but COVID-19-related economic impacts may translate into offsets in demand growth. In the case that these follow the pessimistic end of recovery projections (*29*), this may lead to reductions in anticipated cumulative year 2020–2050 global aviation CO_2_ of around 6%–9% compared with an equivalent case without the pandemic, and 2%–10% reductions in cumulative 2020–2050 airline profit. One impact of COVID-19 is to increase the already large level of uncertainty in relation to future aviation emissions (*28*), highlighting the importance of aviation emissions policy that is responsive to future developments.

The largest-scale current aviation emissions policies are ICAO’s CORSIA and the inclusion of aviation in the EU ETS. We find that COVID-19 leads to zero offsetting requirements in CORSIA’s 2021–2023 pilot phase in around 58% of modeled scenarios, and in 37% of modeled scenarios to zero offsetting requirements in CORSIA’s 2024–2026 first phase. Beyond this timeframe, offsetting requirements are highly uncertain, but we project totals below most current literature estimates because of a combination of long-term participation and demand scenarios and the impact of COVID-19. If the recovery period is extended, ongoing demand growth is low, and/or CORSIA offsets are not fully additional, the aggregate impact of the scheme may be minimal. Because the EU ETS has a much more stringent baseline, we project less uncertainty in ongoing EU ETS allowance requirements. Although European aviation CO_2_ may be close to the EU ETS aviation cap in 2020, it is unlikely to remain below the cap for a significant period. In low demand growth cases the EU ETS, despite its much smaller geographic scope, is responsible for significantly more reductions in net aviation CO_2_ than CORSIA by 2030.

These model runs assume relatively conservative developments in aircraft and fuel technologies, consistent with recent historical trends and some projections of future developments (*9*). Under these assumptions, the aviation industry is unlikely to meet year-2050 targets. Even carbon-neutral growth from 2019, a relatively unambitious target, is missed in 91% of the COVID-19-adjusted probabilistic model runs, and all COVID-19-adjusted scenarios. No model runs meet the IATA target of halving year-2050 aviation CO_2_ relative to year-2005 levels. To meet or go beyond these targets, the reductions in typical demand growth modeled here are not sufficient. The extent to which radical technological change is achievable represents another degree of uncertainty to be explored if the aviation sector is to join others in decarbonizing. This includes the extent to which more ambitious policy on offsets, fuels (including the EC RefuelEU proposals [*68*]) or alternative aircraft technologies can address the potential gap between targets and projections. Similarly, there is a need for future work which explores the extent to which changes in attitudes to aviation may affect ongoing demand for flights, and how this interacts with global and regional income levels by trip purpose, which is a key area of uncertainty in future projections.
